# Perinatal outcomes after a prenatal diagnosis of a fetal copy number variant: a retrospective population-based cohort study

**DOI:** 10.1186/s12887-024-05012-6

**Published:** 2024-08-22

**Authors:** Cecilia Pynaker, Jacqui McCoy, Jane Halliday, Sharon Lewis, David J. Amor, Susan P. Walker, Lisa Hui, On behalf of the PALM cohort study group, Joanne Kennedy, Fiona Norris, Lucy Gugasyan, Emma Brown, Suzanne Svobodova, Matthew Regan, Helen Kincaid, Anand Vasudevan, Susan Fawcett, Melissa Graetz, Joanne Said, Lisa Begg, Nicole Yuen, Natasha Frawley, Geraldine Masson

**Affiliations:** 1 Reproductive Epidemiology Group, Murdoch Children’s Research Institute, Parkville, VIC Australia; 2 Department of Paediatrics, University of Melbourne, Parkville, VIC Australia; 3 Neurodisability and Rehabilitation Group, Murdoch Children’s Research Institute, Parkville, VIC Australia; 4 Department of Obstetrics, Gynaecology and Newborn Health, University of Melbourne, Parkville, VIC Australia; 5 Mercy Hospital for Women, Heidelberg, VIC Australia; 6 Northern Health, Melbourne, VIC Australia

**Keywords:** Prenatal diagnosis, Chromosomal microarray analysis, Copy number variants, Variant of uncertain significance, Perinatal outcomes, Cohort studies, Paediatric population, Feasibility studies

## Abstract

**Background:**

There are no established guidelines for the follow up of infants born after a prenatal diagnosis of a genomic copy number variant (CNV), despite their increased risk of developmental issues. The aims of this study were (i) to determine the perinatal outcomes of fetuses diagnosed with and without a CNV, and (ii) to establish a population-based paediatric cohort for long term developmental follow up.

**Methods:**

An Australian state-wide research database was screened for pregnant individuals who had a prenatal chromosomal microarray (CMA) between 2013–2019 inclusive. Following linkage to laboratory records and clinical referrer details, hospital records were manually reviewed for study eligibility. Eligible participants were mother–child pairs where the pregnancy resulted in a livebirth, the mother was able to provide informed consent in English (did not require a translator) and the mother was the primary caregiver for the child at hospital discharge after birth. Research invitations were sent by registered post at an average of six years after the prenatal diagnostic test. Statistical analysis was performed in Stata17.

**Results:**

Of 1832 prenatal records examined, 1364 (74.5%) mother–child pairs were eligible for recruitment into the follow up cohort. Of the 468 ineligible, 282 (60.3%) had ‘no live pregnancy outcome’ (209 terminations of pregnancy (TOP) and 73 miscarriages, stillbirths, and infant deaths), 157 (33.5%) required a translator, and 29 (6.2%) were excluded for other reasons. TOP rates varied by the type of fetal CNV detected: 49.3% (109/221) for pathogenic CNVs, 18.2% (58/319) for variants of uncertain significance and 3.3% (42/1292) where no clinically significant CNV was reported on CMA. Almost 77% of invitation letters were successfully delivered (1047/1364), and the subsequent participation rate in the follow up cohort was 19.2% (201/1047).

**Conclusions:**

This study provides Australia’s first population-based data on perinatal outcomes following prenatal diagnostic testing with CMA. The relatively high rates of pregnancy loss for those with a prenatal diagnosis of a CNV presented a challenge for establishing a paediatric cohort to examine long term outcomes. Recruiting a mother–child cohort via prenatal ascertainment is a complex and resource-intensive process, but an important step in understanding the impact of a CNV diagnosis in pregnancy and beyond.

**Trial registration:**

ACTRN12620000446965p; Registered on April 6, 2020.

**Supplementary Information:**

The online version contains supplementary material available at 10.1186/s12887-024-05012-6.

## Background

Chromosomal microarray analysis (CMA) can interrogate the human genome to a higher resolution than G-banded karyotyping [1]. This has enabled the detection of submicroscopic deletions and duplications, termed copy number variants (CNVs), which can be benign or pathogenic depending on their location and gene content. CMA is well-established as the gold-standard first-tier diagnostic test for paediatric patients with an unexplained developmental disability, intellectual disability or congenital anomalies, providing 15–20% higher diagnostic yield than G-banded karyotyping [2–4].

Alongside paediatric care, CMA has also revolutionised prenatal care. It has been 10 years since CMA replaced G-banded karyotyping as the recommended diagnostic test for fetuses with an ultrasound abnormality because of its superior diagnostic yield [5–7]. CMAs can detect pathogenic copy number variants (pCNV) linked to established syndromes, but also CNV of uncertain clinical significance (VUS). VUS are CNVs that often involve non-disease causing genes, may not have been previously identified or described, or for which there is limited information on genotype–phenotype correlation due to incomplete penetrance and variable expressivity.

In the Australian state of Victoria, there has been an increase in the proportion of prenatal diagnostic tests analysed with CMA, from 39.4% in 2013 to 93.1% in 2021, regardless of indication for testing [8]. Concurrently, the absolute number of fetuses diagnosed with a pCNV has increased over the past decade, from 25 pCNVs in 2013 to 61 pCNVs in 2022 in the background of a declining number of prenatal diagnostic procedures [8]. The most frequent pCNVs in our population are 22q11.2 deletion (DiGeorge syndrome), 4p16.3 deletion (Wolf-Hischhorn syndrome), and 5p15.33 deletion (cri-du-chat syndrome) [9]. These account for 13.5%, 3.9%, 3.0% of pregnancies with a pCNV respectively. However, the vast majority of pCNVs are rare, which makes counselling on long term childhood outcomes difficult, especially when ascertained before birth when the phenotype is incomplete.

A VUS is diagnosed in approximately 5% of fetuses following chorionic villus sampling or amniocentesis [8]. These VUS can be challenging as there is often no prenatal phenotype to guide CNV interpretation. The limited data available in the literature are commonly skewed towards cases diagnosed postnatally, possibly biased towards the more severe end of the phenotypic spectrum [10–12]. In Australia, there are currently no guidelines for the routine follow up and assessment of children with a prenatally diagnosed VUS. Furthermore, as genomic databases and clinical interpretation guidelines are updated some prenatal VUS are subsequently reclassified as either pathogenic or benign [13]. This highlights the challenge of providing appropriate long term care, as children may not only be lost to follow up after the newborn period, but may also carry a nonextant genetic diagnosis throughout childhood due to changes in scientific knowledge [12, 14].

The PrenatAL Microarray (PALM) is a nationally-funded cohort study of mother–child pairs who have had a prenatal diagnosis with a chromosomal microarray (CMA) from 2013 to 2019 in the Australian state of Victoria. In brief, this cohort study of children- with and without a prenatally-ascertained CNV- aims to examine their developmental, social-emotional and health outcomes in early childhood through a range of parent completed questions, in person cognitive assessments, and clinical paediatric review [15]. The full protocol has been previously published in this journal [15].

In this paper, we report the perinatal outcomes of fetuses that had a prenatal chromosomal microarray (potential PALM participants, with and without a CNV), including rates of termination of pregnancy (TOP) and spontaneous perinatal losses following prenatal diagnosis. We also present the challenges of creating a representative paediatric cohort of children from a prenatal cohort.

## Methods

### Study population

This was a population-based study set in the Australian state of Victoria. Victoria has approximately 78,000 births per year with a median maternal age of 31.5 years [16]. All pregnant individuals are offered screening for fetal structural anomalies and chromosomal conditions, with an uptake of 83.6% state-wide [17]. Between 2013–2021, 80.9% of prenatal diagnostic tests were analysed by CMA [8]. TOP is lawful on maternal request up to 24 weeks, and after 24 weeks if two medical practitioners deem it “appropriate in all the circumstances” [18].

Maternity healthcare in Australia operates through a dual public–private system. Public care, funded by Medicare, offers subsidized services in public hospitals. Private care, covered by private health insurance or out-of-pocket, provides additional options such as choosing obstetricians and amenities in private hospitals. In Australia, approximately 75% of births occur in a public hospital.

### Eligibility criteria for paediatric cohort

Participants were eligible if: the pregnancy resulted in a livebirth, they were the primary-caregiver for the child at hospital discharge, resident in the Australian state of Victoria, and able to provide informed consent in English (did not require a translator).

### Data sources

Multiple sources were utilised to identify and pre-screen potential study participants.

#### Victorian prenatal diagnosis database

The Victorian Prenatal Diagnosis Database (VPDD) is a population-based research dataset that collects all chromosome testing results from amniotic fluid and chorionic villus samples (CVS) [8]. The VPDD was screened for pregnant individuals who underwent prenatal diagnosis with CMA from January 2013 to December 2019. Clinical laboratories classified prenatal CNVs in accordance with the guidelines of the American College of Medical Genetics (ACMG) and other established guidelines [19–21]. CNVs were classified as ‘pathogenic’ when they encompassed a region implicated in a well-described abnormal phenotype, as documented in multiple peer-reviewed publications. ‘Likely pathogenic’ variants were CNVs that met the ACMG definitions of a CNV ‘described in a single case report but with well-defined breakpoints and phenotype, both specific and relevant to the patient findings’, or CNV interval ‘with very compelling gene function that is relevant and specific to the reason for patient referral’. ‘Cases’ included pregnancies with a pathogenic or likely pathogenic CNV (pCNV) and variant of uncertain significance (VUS). ‘Controls’ were pregnancies that had a primary clinical indication other than an ultrasound abnormality and had ‘no clinically significant genomic imbalance’ reported on CMA. These included positive (‘high chance) screening result (non-invasive prenatal testing, combined first trimester screening, or second trimester serum screening), and other testing indications (such as advanced maternal age, maternal request).

Due to the modifier of a structural anomaly or known genetic condition on childhood outcomes, ‘controls’ with a clinical indication of a fetal structural abnormality, family history of a chromosomal condition or a single gene condition were excluded. However, there were some controls where the ultrasound abnormality was a secondary indication after a primary indication of a positive screening result. These were predominantly soft markers (increased nuchal translucency, hypoplastic nasal bone). Further details will be available in the next phase of the PALM study and any controls with a major structural abnormality on antenatal ultrasound excluded from analysis.

Results of single gene testing were not available for the entire cohort. These results were only available for the final consented PALM study participants and will not be reported here.

Clinical laboratories that submitted the cases and controls to the VPDD internally reidentified records and obtained the name of the public maternity hospital or private clinical referrer. Follow up was different for these two groups:

- Public hospital medical record review

Hospital medical records were manually reviewed for perinatal outcome and study eligibility. Perinatal outcomes were coded as either miscarriage (spontaneous pregnancy loss < 20 weeks’ gestation), stillbirth (infant born with no signs of life ≥ 20 weeks’ gestation), TOP, neonatal death (death within 28 days of birth), infant death (death within 2 years of birth), or live birth.

A minimum de-identified dataset was collected for all individuals screened containing: hospital name, maternal postcode, test date, gestational age, clinical indication, CNV classification, perinatal outcome, parity, and study eligibility status. Maternal postcode was mapped to the corresponding local government area and assigned the relevant Index of Relative Socioeconomic Advantage and Disadvantage (IRSAD) allocated by the Australian Bureau of Statistics from 2016 Census data [22].

b) Private clinical referrers

Private clinicians were contacted by phone and/or email and asked to pre-screen their patients for study eligibility and send an invitation letter to eligible participants. No minimum dataset was collected for these patients as pre-screening was performed at the clinician’s discretion.

### Study recruitment

Study invitation letters were sent by registered post between November 2021 and June 2023. Each contained a participant information and consent form, hard-copy questionnaire booklet, and replied paid envelope. Registered post enabled tracking of research letters, including proof of mailing and signature on delivery. The public roll of the Australian Electoral Commission (AEC) was used to check the details of participants whose post were returned to sender. Participants had the option to complete a hard copy or online consent form and questionnaire. Completed hard copies were returned using the provided replied paid envelope, while the online version, hosted in Research Electronic Data Capture (REDCap), were accessed via a QR code in the invitation letter [23, 24].

### Protocol amendments

#### Pandemic impacts

In response to the COVID-19 pandemic in 2020, the study protocol was updated to include alternative online child assessments. Due to pandemic-related disruptions, a 12-month extension was requested due to delays in recruitment, participant assessments, and obtaining approvals for new regional sites.

#### Low recruitment rate

In response to a low recruitment rate, the study protocol was amended. Participants were: (i) sent two reminder letters, and (ii) offered an AUD$110 gift card in appreciation of their time. The first reminder was sent three weeks after the initial study invitation (if successfully delivered), and the second reminder three weeks after the first reminder. Ethics Committee approval for all amendments was obtained.

### Statistical analysis

Statistical analysis was performed in Stata17 using chi-squared test for proportions with *p* < 0.05 considered significant [25]. Wilson score method was used to calculate 95% confidence intervals using Epitools [26].

### Ethics approval

This study received Human Research Ethics Committee approval from the Royal Children’s Hospital on April 8, 2020 (Reference no. 60542) and Mercy Health on September 15, 2020 (Reference no. 2020–046).

## Results

### Pre-screening of potential participants

During the 7-year study period 8184 prenatal diagnostic tests were performed by CMA; a fetal CNV was reported for 1029 samples (12.6%, 95%CI: 11.9–13.3%), and ‘no clinically significant CNV’ reported in 7155 samples (87.4%, 95%CI: 85.7–88.1%). Of these, 4316 with ‘no clinically significant CNV’ were excluded due to an indication of an ultrasound abnormality, family history of a chromosomal condition, or a single gene condition.

**Fig. 1:**
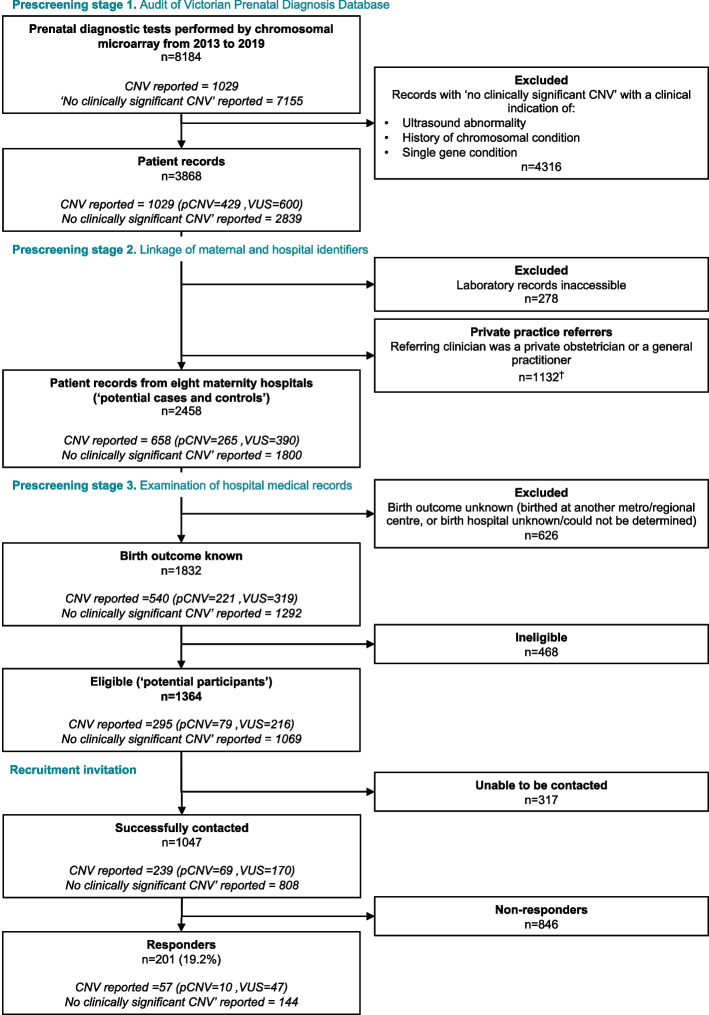
Study flowchart of the pre-screening and recruitment of participants. Abbreviations: CNV, copy number variant; pCNV, pathogenic copy number variant; VUS, copy number variant of uncertain significance. ^†^ In total, 14 participants were recruited into the study through a private obstetrician or general practitioner (data not shown)

Figure 1 illustrates the pre-screening and recruitment of participants with and without a copy number variant.

The personal identifiers of the 2458 potential cases and controls were provided to the eight relevant hospitals and were manually reviewed for eligibility criteria. Only 74.5% (*n* = 1832) had a known birth outcome documented in the hospital records. The remaining missing birth outcome data was due to patients delivering in a different hospital to the one in which the prenatal diagnostic procedure was performed.

Of those with a known birth outcome (*n* = 1832), 1364 (74.5%) were eligible for recruitment (‘potential participants’) and 468 (25.5%) were ineligible. Of the 468 ineligible, 282 (60.3%) had ‘no live pregnancy outcome’ (209 terminations of pregnancy (TOP) and 73 miscarriages, stillbirths, and infant deaths), 157 (33.5%) required a translator, and 29 (6.2%) were excluded for other reasons (Table 1).

**Table 1:** Reasons for study ineligibility

Reason for study ineligibility	Pathogenic copy number variant	Variant of uncertain significance	No clinically significant genomic imbalance	Total
	**n (% pCNVs)**	**n (% VUS)**	**n (% NAD)**	**n (% total)**
Perinatal/infant death	128 (90.1)	75 (72.8)	79 (35.4)	282 (60.3)
Translator required	12 (8.5)	22 (21.4)	123 (55.2)	157 (33.5)
Mother (gestational carrier) not the intended primary caregiver	1 (0.7)	1 (1.0)	7 (3.1)	9 (1.9)
Multifetal pregnancy	0 (0.0)	0 (0.0)	2 (0.9)	2 (0.4)
Unable to provide informed consent	0 (0.0)	1 (1.0)	0 (0.0)	1 (0.2)
Other^a^	1 (0.7)	4 (3.9)	12 (5.4)	17 (3.6)
**Total**	**142 (100.0)**	**103 (100.0)**	**223 (100.0)**	**468 (100.0)**

*Abbreviations*: *pCNV* pathogenic copy number variant, *VUS* variant of uncertain significance, *NAD* no clinically significant genomic imbalance

^a^ Other reasons for study ineligibility were made at the discretion of the site Principle Investigator

### Perinatal outcomes by CMA result

Birth outcomes varied significantly by the type of fetal CNV detected and are presented in Table 2. Fetuses with a pathogenic CNV had a higher TOP rate compared with those with a VUS (49.3% vs. 18.2%, *p* < 0.05) or ‘no clinically significant’ CNV (3.3%, *p* < 0.05).

**Table 2:** Birth outcome of fetuses with and without a copy number variant

Pregnancy result	Pathogenic copy number variant	Variant of uncertain significance	No clinically significant genomic imbalance	Total
	**n (% pCNVs)**	**n (% VUS)**	**n (% NAD)**	**n (% total)**
Livebirth	93 (42.1)	244 (76.5)	1213 (93.9)	1550 (84.6)
Termination of pregnancy	109 (49.3)	58 (18.2)	42 (3.3)	209 (11.4)
Spontaneous stillbirth (≥ 20 weeks)	9 (4.1)	10 (3.1)	19 (1.5)	38 (2.1)
Miscarriage (< 20 weeks)	7 (3.2)	2 (0.6)	10 (0.8)	19 (1.0)
Neonatal death (death within 28 days of birth)	2 (0.9)	4 (1.3)	8 (0.6)	14 (0.8)
Infant death (death within 2 years of birth)	1 (0.5)	1 (0.3)	0 (0.0)	2 (0.1)
**Total**	**221 (100.0)**	**319 (100.0)**	**1292 (100.0)**	**1832 (100.0)**

*Abbreviations*: *pCNV* pathogenic copy number variant, *VUS* variant of uncertain significance, *NAD* no clinically significant genomic imbalance

### Perinatal outcome by indication for prenatal diagnosis

The perinatal outcomes of pregnancies with and without a CNV varied by indication for prenatal diagnosis (Table 3). For pregnancies with a pCNV or VUS the most common indication for prenatal diagnosis was an ultrasound abnormality, 54.8% (121/221) and 62.4% (199/319) respectively. A TOP occurred in 56.2% (68/121) of cases with a pCNV and an ultrasound abnormality. In comparison, when there was a VUS and an ultrasound abnormality, a TOP occurred in 30.6% (61/199) of cases. Almost two percent of pregnancies with ‘no clinically significant genomic imbalance’ and a high chance screening result resulted in a TOP (17/1067). These did not have an ultrasound abnormality.

**Table 3:** Birth outcomes of pregnancies with and without a copy number variant by indication for prenatal diagnosis

Indications for prenatal diagnosis	Livebirth	Termination of pregnancy	Spontaneous stillbirth (≥ 20 weeks)	Miscarriage (< 20 weeks)	Neonatal/infant death	Total
	n (% indication)	n (% indication)	n (% indication)	n (% indication)	n (% indication)	n (% indication)
**Pathogenic copy number variant**						
Ultrasound abnormality^a^	39 (32.2)	68 (56.2)	7 (5.8)	4 (3.3)	3 (2.5)	121 (100.0)
Positive screening result^b^	35 (64.8)	16 (29.6)	1 (1.9)	2 (3.7)	0 (0.0)	54 (100.0)
All other testing indications^c^	19 (41.3)	25 (54.3)	1 (2.2)	0 (0.0)	0 (0.0)	46 (100.0)
**Total**	**93 (42.1)**	**109 (49.3)**	**9 (4.1)**	**7 (3.2)**	**3 (1.4)**	**221 (100.0)**
**Variant of uncertain significance**						
Ultrasound abnormality^a^	138 (68.3)	46 (23.1)	9 (4.5)	2 (1.0)	4 (2.0)	199 (100.0)
Positive screening result^b^	74 (93.7)	3 (3.8)	1 (1.3)	0 (0.0)	1 (1.3)	79 (100.0)
All other testing indications^c^	32 (78.0)	9 (22.0)	0 (0.0)	0 (0.0)	0 (0.0)	41 (100.0
**Total**	**244 (76.5)**	**58 (18.2)**	**10 (3.1)**	**2 (0.6)**	**5 (1.6)**	**319 (100.0)**
**No clinically significant genomic imbalance**						
Ultrasound abnormality^a^	53 (68.8)	18 (23.4)	4 (5.2)	1 (1.3)	1 (1.3)	77 (100.0)^d^
Positive screening result^b^	1024 (96.0)	17 (1.6)	11 (1.0)	8 (0.7)	7 (0.7)	1067 (100.0)
All other testing indications^c^	136 (91.9)	7 (4.7)	4 (2.7)	1 (0.7)	0 (0.0)	148 (100.0)
**Total**	**1213 (93.9)**	**42 (3.3)**	**19 (1.5)**	**10 (0.8)**	**8 (0.6)**	**1292 (100.0)**

^a^Ultrasound abnormality included soft marker on ultrasound such as increased nuchal translucency and hypoplastic nasal bone

^b^Positive (‘high chance’ or ‘high risk’) screening result included non-invasive prenatal testing, first trimester combined screening and second trimester serum screening

^c^All other testing indications” included family history of chromosomal abnormality, single gene condition, and advanced maternal age

^d^70/77 had a concurrent indication of a positive (‘high chance’ or ‘high risk’) screening result

### Responders and non-responders

A total of 3304 research letters were sent to 1364 eligible patients over the 20-month study period (1607 invitations (including repeats), 893 first reminders, 804 s reminders) (Fig. 1). Initially, a third of all study invitations were returned to sender due to incorrect address (31.9%, 435/1364). An updated address was found for 43.2% (188/435) of these using the AEC public electoral roll. Overall, 1047 participants were successfully contacted (1047/1364, 76.8%).

The rate of informed consent to participate in the PALM study among those who were successfully contacted (‘responders’) was 19.2% (201/1047) (Fig. 1).

### Comparison of cases and controls

Data collected through examination of hospital medical records were used to compare the 1832 ‘potential participants’ (with a known birth outcome) stratified by CNV status (cases vs. controls).

There were no consistent patterns of difference in sociodemographic characteristics between cases and controls in their study eligibility or ability to be contacted via mail (see Supplementary Table 1 and Supplementary Table 2 for details). A slight difference in response rate was observed with 23.8% of cases responding compared with 17.8% of controls (Table 4).

**Table 4:** Potential sources of participation bias

	**Cases**			**Controls**		
**Variable**	**Responders (participants)**	**Non-responders**	***P* value**	**Responders (participants)**	**Non-responders**	***P* value**
	***n* = 57 (%)**	***n* = 182 (%)**		***n* = 144 (%)**	***n* = 664 (%)**	
**IRSAD quintile**						
1 (most disadvantaged)	9 (15.8)	29 (15.9)	0.40	6 (4.2)	75 (11.3)	0.012
2	4 (7.0)	25 (13.7)		22 (15.3)	77 (11.6)	
3	13 (22.8)	52 (28.6)		29 (20.1)	184 (27.7)	
4	17 (29.8)	46 (25.3)		48 (33.3)	190 (28.6)	
5 (most advantaged)	14 (24.6)	20 (16.5)		39 (27.1)	138 (20.8)	
**Remoteness area**						
Metropolitan	45 (78.9)	158 (86.8)	0.15	127 (88.2)	613 (92.3)	0.11
Regional/remote	12 (21.1)	24 (13.2)		17 (11.8)	51 (7.7)	
**Mother’s age at recruitment**						
35 years	13 (22.8)	48 (26.4)	0.31	8 (5.6)	67 (10.1)	0.24
35 – 39 years	20 (35.1)	45 (24.7)		35 (24.3)	155 (23.3)	
≥ 40 years	24 (42.1)	89 (48.9)		101 (70.1)	442 (66.6)	
**Parity**						
0	22 (38.6)	58 (31.9)	0.83	55 (38.2)	205 (30.9)	0.0042
1	21 (36.8)	68 (37.4)		50 (34.7)	251 (37.8)	
2	9 (15.8)	37 (20.3)		30 (20.8)	115 (17.3)	
3	3 (5.3)	14 (7.7)		7 (4.9)	43 (6.5)	
4 +	2 (3.5)	5 (2.7)		0 (0.0)	34 (5.1)	
Missing	0 (0.0)	0 (0.0)		2 (1.4)	16 (2.4)	
**Child’s age at recruitment**						
≥ 5 years	25 (43.9)	58 (31.9)	0.097	35 (24.3)	173 (26.1)	0.66
5 years	32 (56.1)	124 (68.1)		109 (75.7)	491 (73.9)	

*IRSAD* Index of Relative Socioeconomic Advantage and Disadvantage

Compared with non-responding controls, participating controls were significantly more likely to be of higher socioeconomic status and lower parity. There was no evidence of any differences between responders and non-responding cases (Table 4).

#### Private practice referrers

A substantial proportion of patients were referred for their diagnostic procedure by a private referrer rather than a public hospital (1132/3868, 29.3%, Fig. 1). These 1132 patients were referred by 497 private clinicians. Of these, 304 clinicians (associated with 874 patients) could be contacted by phone and/or email; 28 clinicians (9.2%; associated with 119 patients (13.6%)) agreed to pre-screen their patients for study eligibility; 14 patients were ultimately recruited through this method. Data from the 119 patients screened by private referrers were not collected and were excluded from this analysis as pre-screening of potential participants was performed at the discretion of the private referrer.

#### Regional participants

One in five patients who had a prenatal diagnostic procedure in a tertiary hospital was referred from a regional hospital (175/850, 20.6%). To assist recruitment and minimise selection bias by location, the protocol was amended to include data collection from the three largest referring regional health services (representing 156/175 regional patients). It took on average 20-months from ethics amendment approval to site governance approval. The additional burden of adding these three sites resulted in an additional 9 participants (all cases).

#### Recruitment rate

Overall, the 201 mother–child pairs were recruited into the PALM childhood outcome study, comprising 144 controls and 57 cases (10 pCNVs, 47 VUS). This cohort represented 2.5% of the initial 8184 prenatal diagnosis cases pre-screened from the Victorian Prenatal Diagnosis Data Collection. This was lower than 8.7% (719/8184) recruitment rate estimated in the original study protocol [15].

## Discussion

### Principle findings

CMA has been instrumental in improving the diagnostic yield of prenatal diagnosis, but published data on the perinatal and paediatric outcomes of fetal CNVs are scarce due to their rarity. We conducted a lengthy and thorough manual linkage process to retrospectively recruit 201 mother–child pairs for a prospective childhood outcome study from a starting data source of re-identifiable prenatal diagnostic test results. This complex process was necessary to overcome the challenges of maintaining patient privacy and traversing siloed medical record information across health services and research institutes.

### Results in the context of what is known

Overall, the rates of TOP for the pCNVs in our cohort were at the lower end of the range reported in the literature. We observed a 49.3% TOP rate for pCNVs overall and a 56.2% TOP rate for pCNVs with an ultrasound abnormality. This compares with TOP rates for pCNV ranging from 50–100% in other studies [13, 14, 27–32]. Higher rates have been report for pCNVs with an ultrasound abnormality (either structural or soft marker) (76.2–100%) in China, France, and Israel [13, 29–32].

Similarly, for VUS we observed a 18.2% TOP rate for VUS overall and a 23.1% rate for VUS with an ultrasound abnormality. Again, this aligns with the lower end of reported TOP rates for VUS from 11.0–44.9% [14, 29–31, 33, 34], with higher rates for de novo VUS (50.8–58.0%) [13, 34] and fetuses with an ultrasound abnormality (17.1–37.5%) [13, 29–31, 34]. There are many methodological factors that may contribute to the different TOP rates for pCNV and VUS that preclude meaningful direct comparisons between studies. These factors include highly variable cohort sizes, study inclusion criteria, clinical testing pathways, CNV classification systems, health system and patient factors, availability of TOP, and timing of prenatal diagnosis.

### Clinical and research implications

Paediatrics cohorts established from prenatal genomic testing populations are rare due to logistical and ethical challenges, yet they are crucial for obtaining long-term outcome data. A previous cohort of this nature examined the childhood outcomes of children prenatally diagnosed with confined placental mosaicism in the Australian state of Victoria [35]. By utilising the same prenatal diagnosis dataset this paediatric cohort was not biased towards children with an established clinical phenotype. Despite contacting participants 5.5 years after prenatal diagnosis, the study achieved a recruitment rate of 76%. This is almost four times the response rate achieved in our study (19.1%). It is unknown what factors contributed to the higher recruitment rate but one factor could be the clinician-patient relationship as the treating doctor facilitated the contact between the participant and the research team, rather than a hospital departmental representative as in our study. Moreover, despite modifying the study protocol to minimise exposure to COVID-19 (such as offering virtual assessments), the pandemic might have negatively influenced patient attitudes towards participating in research studies [36].

There are only two other studies that have reported paediatric outcomes of children with a prenatal diagnosis of a CNV [13, 37]. Shi et al. prospectively followed up 109 children with a prenatally diagnosed VUS up the age of 2–4 years. Five children apparently showed clinical signs or phenotypic features of disease, but the clinical assessments were not described in detail. Muys et al. retrospectively recruited 85 mother–child pairs with a pCNV or VUS (‘cases’) and 123 with no or a benign CNV (‘controls’). The response rate of 15.8% (208/1312) was lower than our results, even though their study had lower participant burden (parental questionnaire only). However, these response rates may not be comparable. Muys et al. did not perform extensive pre-screening to determine perinatal outcomes: several participants were ineligible due to a perinatal or neonatal death. Had we not performed our extensive pre-screening step, 282 women who experienced a perinatal loss would have been invited to participate in our childhood outcome study. Also, Muys et al. did not report the number of research invitation letters successfully delivered, only the total number sent. Use of registered post and manual follow up at the AEC public roll enabled us to determine the number of potential participants successfully contacted. Of note 21.3% (43/201) of our final cohort were contacted following use of the AEC to update contact addresses, demonstrating the value of this additional step.

### Strengths and limitations

This is one of the largest studies to manually determine the perinatal outcomes of pregnancies with and without a prenatally diagnosed CNV. Through this process, we have successfully established a paediatric cohort and prospectively conducted detailed cognitive and clinical assessments on each child. Data analysis is currently underway, with public dissemination of results expected in 2024.

Another one of our strengths was the attention paid to patient psychological safety through manual record review and the exclusion of patients who had experienced a perinatal loss or infant death. We thereby averted potential distress for 282 families by removing them from our study invitation list. However, the extensive pre-screening procedures at multiple maternity hospitals and the siloed nature of Australian health records pose substantial resource and administrative barriers to future research of this type.

One of the limitations of our cohort is the missing birth data on one quarter of potentially eligible patients due to private and regional referral patterns. This finding highlights the challenges in tracing participants and engaging with hospitals for population-based research studies.

Another limitation of this study is that our ethics approval did not permit us to retain data on perinatal outcomes by specific CNVs due privacy concerns with potentially identifying information. We have previously reported the most common CNVs in our population from 2012 to 2018 [9]. The three most frequent pathogenic CNVs were 22q11.2 deletion syndrome, 4p16.3 deletion, and 5p15.33 deletion. The three most frequent VUS were 15q11.2 del, 22q11.2 duplication and 1q21.1 duplication. Further details including perinatal outcomes and ultrasound abnormalities are available in that publication.

### Future directions

Establishing systems that enable the routine paediatric follow up of prenatally diagnosed genetic abnormalities are essential for understanding the full phenotypic spectrum of CNVs. In particular, there are no standard recommendations regarding long term follow up of children with VUS. Our cohort will provide long term outcome data on the developmental outcomes of children with VUS that may help guide future clinical care.

One of the barriers to collecting long term outcomes from genomic CNVs is the limitations of current congenital anomaly coding systems such as the International Classification of Diseases 10^th^ Revision (ICD-10). The ICD-10 has very few specific genetic diagnostic codes as it is based on phenotypes and organ systems. More locally, our state-wide Victorian Congenital Anomalies Report reports the population prevalence and birth outcomes for common autosomal trisomies but not CNVs (pathogenic or VUS) [38]. Expanding the reporting of congenital anomalies to encompass a broader spectrum of genomic variants would offer a more comprehensive understanding of the impact of prenatal diagnosis and enable us to perform better quality linkage studies for long term outcome data.

## Conclusion

This study provides Australia’s first population-based data on perinatal outcomes including termination of pregnancy following prenatal diagnostic testing with CMA. Three-quarters of fetuses with a VUS and less than half of fetuses with a pCNV resulted in a livebirth. Our establishment of a mother–child cohort via prenatal ascertainment was a complex and resource-intensive process, but an important step in understanding the impact of a CNV diagnosis in pregnancy and beyond.

### Supplementary Information

Supplementary Material 1: Supplementary Table 1. Sociodemographic characteristics of eligible and ineligible participants. IRSAD, Index of Relative Socioeconomic Advantage and Disadvantage.

Supplementary Material 2: Supplementary Table 2. Sociodemographic characteristics of cases and controls by ability to be contacted by mail. IRSAD, Index of Relative Socioeconomic Advantage and Disadvantage.

## Data Availability

The data that support the findings of this study are available from the corresponding author to researchers from a recognised academic institution upon reasonable request.

## Abbreviations

AEC: Australian Electoral Commission
CMA: Chromosomal microarray
CNV: Copy number variant
CVS: Chorionic villus sampling
HREA: Human Research Ethics Application
HREC: Human Research Ethics Committee
MCRI: Murdoch Children’s Research Institute
NHMRC: National Health and Medical Research Council
PALM: PrenatAL Microarray
pCNV: Pathogenic copy number variant
TOP: Termination of pregnancy
VPDD: Victorian Prenatal Diagnosis Data collection
VUS: Variants of uncertain/unknown significance
