# Supplementary material for: Perinatal outcomes after a prenatal diagnosis of a fetal copy number variant: a retrospective population-based cohort study
Source: BMC Pediatr. 2024 Aug 22;24:536. doi: 10.1186/s12887-024-05012-6 (PMC11340052; doi:10.1186/s12887-024-05012-6)
Supplement: Supplementary file 1 — Supplementary Material 1: Supplementary Table 1. Sociodemographic characteristics of eligible and ineligible participants. IRSAD, Index of Relative Socioeconomic Advantage and Disadvantage. [file 12887_2024_5012_MOESM1_ESM.docx]

**Supplementary Table 1.** Sociodemographic characteristics of eligible and ineligible participants

|  | **Cases** | | | **Controls** | | |
| --- | --- | --- | --- | --- | --- | --- |
| **Variable** | **Eligible** | **Ineligible** | **P value** | **Eligible** | **Ineligible** | **P value** |
|  | **n=245 (%)** | **n=295 (%)** |  | **n=223 (%)** | **n=1069 (%)** |  |
| **IRSAD quintile** | | | | | | |
| 1 (most disadvantaged) | 35 (14.3) | 43 (14.6) | 0.10 | 54 (24.2) | 123 (11.5) | <0.001 |
| 2 | 27 (11.0) | 36 (12.2) |  | 28 (12.6) | 140 (13.1) |  |
| 3 | 45 (18.4) | 80 (27.1) |  | 47 (21.1) | 266 (24.9) |  |
| 4 | 76 (31.0) | 80 (27.1) |  | 57 (25.6) | 300 (28.1) |  |
| 5 (most advantaged) | 62 (25.3) | 56 (19.0) |  | 37 (16.6) | 240 (22.5) |  |
| **Remoteness area** | | | | | | |
| Metropolitan | 212 (86.5) | 248 (84.1) | 0.72 | 211 (94.6) | 954 (89.2) | 0.044 |
| Regional/remote | 33 (13.5) | 47 (15.9) |  | 12 (5.4) | 115 (10.8) |  |
| **Mother’s age at recruitment** | | | | | | |
| < 35 years | 67 (27.3) | 76 (25.8) | 0.41 | 41 (18.4) | 127 (11.9) | 0.014 |
| 35 – 39 years | 77 (31.4) | 81 (27.5) |  | 57 (25.6) | 250 (23.4) |  |
| ≥ 40 years | 101 (41.2) | 138 (46.8) |  | 125 (56.1) | 652 (61.0) |  |
| **Parity** | | | | | | |
| 0 | 109 (44.5) | 98 (33.2) | 0.001 | 68 (30.5) | 353 (33.0) | 0.52 |
| 1 | 83 (33.9) | 108 (36.6) |  | 79 (35.4) | 381 (35.6) |  |
| 2 | 34 (13.9) | 57 (19.3) |  | 43 (19.3) | 198 (18.5) |  |
| 3 | 6 (2.4) | 22 (7.5) |  | 13 (5.8) | 75 (7.0) |  |
| 4+ | 7 (2.9) | 10 (3.4) |  | 15 (6.7) | 42 (3.9) |  |
| Missing | 6 (2.4) | 0 (0.0) |  | 5 (2.2) | 20 (1.9) |  |
| **Child’s age at recruitment** | | | | | | |
| ≥ 5 years | 100 (40.8) | 98 (33.2) | 0.068 | 54 (24.2) | 290 (27.1) | 0.37 |
| < 5 years | 145 (59.2) | 197 (66.8) |  | 169 (75.8) | 779 (72.9) |  |

IRSAD, Index of Relative Socioeconomic Advantage and Disadvantage
