# Supplementary material for: Perinatal outcomes after a prenatal diagnosis of a fetal copy number variant: a retrospective population-based cohort study
Source: BMC Pediatr. 2024 Aug 22;24:536. doi: 10.1186/s12887-024-05012-6 (PMC11340052; doi:10.1186/s12887-024-05012-6)
Supplement: Supplementary file 2 — Supplementary Material 2: Supplementary Table 2. Sociodemographic characteristics of cases and controls by ability to be contacted by mail. IRSAD, Index of Relative Socioeconomic Advantage and Disadvantage. [file 12887_2024_5012_MOESM2_ESM.docx]

**Supplementary Table 2.** Sociodemographic characteristics of cases and controls by ability to be contacted by mail.

|  | **Cases** | | | **Controls** | | |
| --- | --- | --- | --- | --- | --- | --- |
| **Variable** | **Successfully contacted** | **Unable to be contacted** | **P value** | **Successfully contacted** | **Unable to be contacted** | **P value** |
|  | **n=200 (%)** | **n=95 (%)** |  | **n=683 (%)** | **n=386 (%)** |  |
| **IRSAD quintile** | | | | | | |
| 1 (most disadvantaged) | 30 (15.0) | 13 (13.7) | 0.65 | 66 (9.7) | 57 (14.8) | 0.068 |
| 2 | 27 (13.5) | 9 (9.5) |  | 87 (12.7) | 53 (13.7) |  |
| 3 | 56 (28.0) | 24 (25.3) |  | 183 (28.6) | 83 (21.5) |  |
| 4 | 53 (26.5) | 27 (28.4) |  | 195 (28.6) | 105 (27.2) |  |
| 5 (most advantaged) | 34 (17.0) | 22 (23.3) |  | 152 (22.3) | 88 (22.8) |  |
| **Remoteness area** | | | | | | |
| Metropolitan | 171 (85.5) | 77 (81.1) | 0.50 | 625 (91.5) | 329 (85.2) | 0.006 |
| Regional/remote | 29 (14.5) | 18 (18.9) |  | 58 (8.5) | 57 (14.8) |  |
| **Mother’s age at recruitment** | | | | | | |
| < 35 years | 49 (24.5) | 27 (28.4) | 0.40 | 60 (8.8) | 67 (17.4) | <0.001 |
| 35 – 39 years | 52 (26.0) | 29 (30.5) |  | 156 (22.8) | 94 (24.4) |  |
| ≥ 40 years | 99 (49.5) | 39 (41.1) |  | 467 (68.4) | 225 (58.3) |  |
| **Parity** | | | | | | |
| 0 | 70 (35.0) | 28 (29.5) | 0.27 | 215 (31.5) | 138 (35.8) | 0.17 |
| 1 | 77 (38.5) | 31 (32.6) |  | 255 (37.3) | 216 (32.6) |  |
| 2 | 36 (18.0) | 21 (22.1) |  | 127 (18.6) | 71 (18.4) |  |
| 3 | 11 (5.5) | 11 (11.6) |  | 42 (6.1) | 33 (8.5) |  |
| 4+ | 6 (3.0) | 4 (4.2) |  | 32 (4.7) | 10 (2.6) |  |
| Missing | 0 (0.0) | 0 (0.0) |  | 12 (1.8) | 8 (2.1) |  |
| **Child’s age at recruitment** | | | | | | |
| ≥ 5 years | 68 (34.0) | 30 (31.6) | 0.68 | 186 (27.2) | 104 (26.9) | 0.92 |
| < 5 years | 132 (66.0) | 65 (68.4) |  | 497 (72.8) | 282 (73.1) |  |

IRSAD, Index of Relative Socioeconomic Advantage and Disadvantage
